# Modelling and Analysis of the Epidemic Model under Pulse Charging in Wireless Rechargeable Sensor Networks

**DOI:** 10.3390/e23080927

**Published:** 2021-07-21

**Authors:** Guiyun Liu, Ziyi Huang, Xilai Wu, Zhongwei Liang, Fenghuo Hong, Xiaokai Su

**Affiliations:** School of Mechanical and Electric Engineering, Guangzhou University, Guangzhou 510006, China; hzy1026931135@163.com (Z.H.); wxl1379794788@163.com (X.W.); hfhhfh345@163.com (F.H.); Sxk1347623210@163.com (X.S.)

**Keywords:** wireless rechargeable sensor networks, comparison theorem, Floquet theorem, persistence

## Abstract

With the development of wireless sensor networks (WSNs), energy constraints and network security have become the main problems. This paper discusses the dynamic of the Susceptible, Infected, Low-energy, Susceptible model under pulse charging (SILS-P) in wireless rechargeable sensor networks. After the construction of the model, the local stability and global stability of the malware-free T-period solution of the model are analyzed, and the threshold $R_{0}$ is obtained. Then, using the comparison theorem and Floquet theorem, we obtain the relationship between $R_{0}$ and the stability. In order to make the conclusion more intuitive, we use simulation to reveal the impact of parameters on $R_{0}$. In addition, the paper discusses the continuous charging model, and reveals its dynamic by simulation. Finally, the paper compares three charging strategies: pulse charging, continuous charging and non-charging and obtains the relationship between their threshold values and system parameters.

## 1. Introduction

With the rapid development of Internet of Things technology in recent years, more and more scholars have focused on wireless sensor networks (WSNs). WSNs consist of many cheap wireless sensor nodes that consume power. Sensor nodes have acquisition, processing, control and communication functions, making WSNs widely used in various fields, such as collaborative detection of multiple unmanned aerial vehicles and fault diagnosis.

Sensor nodes use a multi-hop or single-hop mode to access the network through the data transmission link. No matter what kind of communication mode, a wireless link is needed for data transmission. It is very difficult to build a perfect security mechanism based on a wireless link. Therefore, the security of WSNs has attracted much attention.

The most fundamental reason that malware can spread in WSNs is the connectivity of the network. Since wireless links are used for data transmission among nodes, it is difficult to construct complex protection mechanisms. In order to curb the spread of malware among nodes, a research team has proposed the weight adaptation scheme [1]. The weight adaptation scheme can block the transmission of malware by reducing the transmission efficiency among nodes. In addition, with the development of WSNs, there has been a lot of studies on network security in recent years and some relevant literature is listed in Table 1. 

At present, the research on network security is mostly based on the perspective of algorithms [10,11,12]. However, there are many perspectives on the research of network security, and it is also a direction that can combine the spread of malicious software with the dynamics of infectious diseases. Since the spread of malware is somewhat similar to the spread of biological viruses, the research on WSNs security can be carried over into the dynamics of infectious diseases [13]. For example, Wang et al. [14] proposed an effective and efficient immunization strategy for MWSNs based on pulse differential equations and the SIR model. Similarly, Liu et al. proposed an optimal control scheme based on the novel epidemic model (SILS) [15], and Cao et al. obtained the optimal control variables of immunization ratio and recovery ratio by using Pontryagin’s maximum principle based on the theory of infectious disease [16]. 

However, up to now, there have been few studies on the security of WSNs using impulse differential equation theory. Therefore, based on the dynamics of infectious diseases, this paper uses the impulse differential equation theory to study the node persistence and network security of WRSNs.

In addition to network security, energy constraints are also an important problem that restricts the development of WSNs [17]. With the development of WSNs, rechargeable technology is also a research trend [18,19,20]. In this paper, the residual energy of nodes and the concept of pulse charging are introduced. Because the charging time is relatively short in the whole network cycle, the charging behavior can be approximated as an instantaneous behavior. Compared with continuous charging, pulse charging is more energy saving. Thus, it is of great significance to study the application of pulse charging in WRSNs. 

The main purpose of this study is to extend the existing network model [21] and verify the stability of disease-free periodic solutions and the persistence of disease. We improved the charging strategy of the existing model to make it more energy efficient. In the next section, we propose the improved version of the SILS model under pulse charging [21]. In Section 3, the stability of the periodic solution is proved by the next generation matrix and Floquet theory. In Section 4 we discuss the persistence of malware transmission. The persistence of malware transmission refers to the persistent spread of malware in WRSNs under certain conditions. The persistence theory of malware transmission is of great significance for study of the cyberspace security problem. In the last section, we verify the accuracy of the theory through simulation.

## 2. Epidemic Modeling

### 2.1. Epidemic Model under Continuous Charging Based on WSNs

Far-field charging is a widely used charging strategy, but this charging strategy is relatively inefficient, so it is often used in low-energy wireless systems such as RFID and WSNs [22]. The following is the low-energy wireless system model built based on far-field charging and epidemic model.

For briefness, sensor nodes are divided into five established compartments: Infected (I), Susceptible (S), low-energy infection (LI), low-energy susceptible to infection (LS), and hardware is damaged (D). Malware attacks the sensor nodes in S state with high probability. The sensor nodes in I state are partially disabled due to malware attacks and perform malicious operations. The low-energy sensor nodes LS and LI are forced to sleep due to energy constraints. The sensor node in the D state is completely disabled due to irreparable hardware damage. Specifically, a sensor node in the dormant state cannot perform data transfer. Thus, low-energy infected nodes in LI cannot spread malware. 

When the malware starts to run, the susceptible nodes in S enter the communication range of the infected nodes in I and are attacked by malware. Because the infected node is not familiar with the network topology, there is a data transfer coefficient for the spread of the malware [16]. Transforming from susceptible nodes to infected nodes is produced with *a*_2_*I*(*t*)*S*(*t*) and the positive number $\alpha_{2}$ is used to denote the data transfer coefficient. The number of sensor nodes in S depends partly on Λ, where Λ is the birth rate. Some infected nodes become susceptible nodes at the conversion rate of $a_{1}$, which is the repair rate. Both of the high-energy nodes I and S are transformed at a rate μ into the low-energy nodes LI and LS. To reduce the complexity of the model, the constant C is used to denote the charging rate. It is assumed that all the charging rates are identical and invariant. Similarly, it assumes that all sensor nodes have the same mortality rate γ. Based on existing studies [21], the SILS model can be expressed as follows

(1a) $$\dot{S\left(t\right)}=\Lambda -\left[\alpha_{2}I\left(t\right)+\mu +\gamma \right]S\left(t\right)+\alpha_{1}I\left(t\right)+CLS\left(t\right)$$

(1b) $$\dot{I\left(t\right)}=\left[-\alpha_{1}-\mu -\gamma +\alpha_{2}S\left(t\right)\right]I\left(t\right)+CLI\left(t\right)$$

(1c) $$\dot{LI\left(t\right)}=-\left(C+\gamma \right)LI\left(t\right)+\mu I\left(t\right)$$

(1d) $$\dot{LS\left(t\right)}=-\left(C+\gamma \right)LS\left(t\right)+\mu S\left(t\right)$$

(1e) $$\dot{D\left(t\right)}=\gamma \left[S\left(t\right)+I\left(t\right)+LS\left(t\right)+LI\left(t\right)\right]$$

Moreover, N(t)=S(t)+I(t)+LS(t)+LI(t), and is constrained by

(1f) $$\dot{N\left(t\right)}=\Lambda -\gamma N\left(t\right)\;\left(1f\right)$$

### 2.2. A Pulse Charging Model for SILS

WSNs with a low duty cycle can maintain permanent operation within a certain range of RF power density, and a pulse charging strategy can accomplish this purpose [22].

By introducing the impulse differential equation into the SILS model [21], we can achieve the SILS model of pulse charging (SILS-P) at different moments. The SILS model discusses a WSNs model of continuous charging while the SILS-P model proposes a new charging strategy based on the same WSNs model and reformulates the system.

In the SILS-P model, charging does not occur continuously but over a period of time that is much smaller than the cycle, which is why charging is seen as a pulse. As t=nT(n=1, 2, 3…), the SILS-P model can be written as follows, which is used to describe the dynamic changes of nodes during charging.

(2) $$\{\begin{array}{c} S\left(t^{+}\right)=S\left(t\right)+CLS\left(t\right)\\ I\left(t^{+}\right)=I\left(t\right)+CLI\left(t\right)\\ LI\left(t^{+}\right)=\left(1-C\right)LI\left(t\right)\\ LS\left(t^{+}\right)=\left(1-C\right)LS\left(t\right)\\ N\left(t^{+}\right)=N\left(t\right) \end{array}$$

where T is the pulse charging period, and $nT^{+}$ is used to represent the next instant of nT. Because the time of pulse charging is much less than one cycle, pulse charging can be regarded as instantaneous behavior. In a nutshell, pulse charging is the charging of low-power nodes at a series of time points (t=nT). When t≠nT(n=1, 2, 3…), the pulse charging model is governed by following Equations (2) and (3) does not consider dynamic changes caused by charging during this period.

(3) $$\{\begin{array}{c} \dot{S\left(t\right)}=\Lambda -\alpha_{2}I\left(t\right)+\mu +\gamma S\left(t\right)+\alpha_{1}I\left(t\right)\\ \dot{I\left(t\right)}=\left[-\alpha_{1}-\mu -\gamma +\alpha_{2}S\left(t\right)\right]I\left(t\right)\\ \dot{LI\left(t\right)}=-\gamma LI\left(t\right)+\mu I\left(t\right)\\ \dot{LS\left(t\right)}=-\gamma LS\left(t\right)+\mu S\left(t\right)\\ \dot{D\left(t\right)}=\gamma \left[S\left(t\right)+I\left(t\right)+LS\left(t\right)+LI\left(t\right)\right] \end{array}$$

The existence of disease-free periodic solutions is the periodic solution of *T* that satisfies the above system of equations when I=0. Since I = 0, we begin the analysis of Equations (2) and (3) by demonstrating the existence of disease-free periodic solutions, and we can obtain the system as follows [23]

(4) $$\{\begin{array}{c} \begin{array}{c} S\left(t^{+}\right)=S\left(t\right)+CLS\left(t\right)\\ LS\left(t^{+}\right)=\left(1-C\right)LS\left(t\right)\\ N\left(t^{+}\right)=N\left(t\right) \end{array}\}\;\;\;t=nT\left(n=1,\;2,\;3\ldots \right)\\ \begin{array}{c} \dot{\;S\left(t\right)}=\Lambda -\left(\mu +\gamma \right)S\left(t\right)\\ \dot{LS\left(t\right)}=-\gamma LS\left(t\right)+\mu S\left(t\right)\\ \dot{N\left(t\right)}=\Lambda -\gamma N\left(t\right) \end{array}\}\;t\neq nT\left(n=1,\;2,\;3\ldots \right) \end{array}$$

When n is a natural number, [nT,(n+1)T] is the time interval between two pulse charges, and the pulse charges at times nT and (n+1)T.

From the last equation of Equations (2) and (1f), we obtain

(5) $$\underset{t\to \infty }{lim}\;N\left(t\right)=\frac{\Lambda }{\gamma }$$

If there is a disease-free periodic solution, then LS(t)=I(t)=0 when t→∞. According to Equation (5), we can obtain the following limit results from system (4)

(6) $$S\left(t\right)=\frac{\Lambda }{\gamma }-LS\left(t\right)$$

In this case, LS and S satisfy the following impulse differential system

(7) $$\{\begin{array}{c} \begin{array}{c} S\left(t^{+}\right)=\left(1-C\right)S\left(t\right)+\frac{\Lambda C}{\gamma }\\ LS\left(t^{+}\right)=\left(1-C\right)LS\left(t\right) \end{array}\}\;\;\;\;\;\;\;\;t=nT\left(n=1,\;2,\;3\ldots \right)\\ \begin{array}{c} \dot{LS\left(t\right)}=\Lambda -\left(\mu +\gamma \right)S\left(t\right)\\ \dot{LS\left(t\right)}=-\left(\gamma +\mu \right)LS\left(t\right)+\frac{\Lambda \mu }{\gamma } \end{array}\}\;\;\;t\neq nT\left(n=1,\;2,\;3\ldots \right) \end{array}$$

The solution of the LS on the interval [nT,(n+1)T] is as follows

(8) $$LS\left(t\right)=\frac{\mu \Lambda }{\left(\mu +\gamma \right)\gamma }+\left(LS\left(nT^{+}\right)-\frac{\mu \Lambda }{\left(\mu +\gamma \right)\gamma }\right)e^{-\left(\mu +\gamma \right)\left(t-nT\right)}$$

Let $LS_{n+1}=LS\left(\left(n+1\right)T^{+}\right)$, using stroboscopic mapping, we can derive the functional relation $LS_{n+1}=f\left(LS_{n}\right)$. The relationship is as follows

(9) $$LS_{n+1}=\left(1-C\right)\left[\frac{\mu \Lambda }{\left(\mu +\gamma \right)\gamma }+\left(LS_{n}-\frac{\mu \Lambda }{\left(\mu +\gamma \right)\gamma }\right)e^{-\left(\mu +\gamma \right)T}\right]$$

Equation (9) has a mapping f, such that $LS_{n+1}=f\left(LS_{n}\right)$. $LS_{n+1}=LS_{n}$ can be determined when Equation (9) is in equilibrium. Thus, Equation (9) has the equilibrium state as follows

(10) $$LS^{*}=\frac{\left(1-C\right)\frac{\mu \Lambda }{\gamma \left(\mu +\gamma \right)}\left(1-e^{-\left(u+\gamma \right)T}\right)}{1-\left(1-C\right)e^{-\left(\mu +\gamma \right)T}}$$

$LS^{*}$ is the point of cyclic convergence of LS(t) at $t_{n}=nT$ with T as the period. Taking the positive equilibrium $LS^{*}$ as the initial value of Equation (9), it is obvious that

(11) $${\left|\frac{df\left(LS_{n}\right)}{dLS}\right|}_{LS=LS^{*}}<1$$

Thus, by using the stability criterion of differential systems, the equilibrium state $LS^{*}$ of Equation (9) is locally stable, which implies the global stability of $LS^{*}$. This means that the sequence $LS_{n}$ will converge to the equilibrium state $LS^{*}$. Thus, we can obtain the periodic solution of Equation (7) as follows

(12) $$\tilde{LS}\left(t\right)=\frac{\mu \Lambda }{\gamma \left(\gamma +u\right)}+\left(LS^{*}-\frac{\mu \Lambda }{\gamma \left(\gamma +u\right)}\right)e^{-\left(\mu +\gamma \right)\left(t-nT\right)}$$

According to Equation (9), we can obtain the equilibrium state of $S_{n+1}=\frac{\Lambda }{\gamma }-\left(1-C\right)\left[\frac{\mu \Lambda }{\left(\mu +\gamma \right)\gamma }+\left(LS_{n}-\frac{\mu \Lambda }{\left(\mu +\gamma \right)\gamma }\right)e^{-\left(\mu +\gamma \right)T}\right]$ as follows

(13) $$S^{*}=\frac{\Lambda }{\gamma }-\frac{\left(1-C\right)\frac{\mu \Lambda }{\gamma \left(\mu +\gamma \right)}\left(1-e^{-\left(u+\gamma \right)T}\right)}{1-\left(1-C\right)e^{-\left(\mu +\gamma \right)T}}$$

In the same way, it is obvious that the sequence $S_{n}$ will converge to the equilibrium state $S^{*}$. According to Equation (6), we can obtain the periodic solution of Equation (7) as follows

(14) $$\tilde{S}\left(t\right)=\frac{\Lambda }{\left(\gamma +u\right)}-\left(S^{*}-\frac{\mu \Lambda }{\gamma \left(\gamma +u\right)}\right)e^{-\left(\mu +\gamma \right)\left(t-nT\right)}$$

## 3. Stability of a Malware-Free T-Period Solution

In this section, the local stability and global stability of the SILS-P model are analyzed.

**Theorem** **1.** *When* $R_{0}<1$ *, the disease-free periodic solution of the system is locally asymptotically stable.*

**Proof.**  For the sake of calculation, let Q(t) be a square matrix of order n. Let $\Phi_{Q}\left(t\right)$ be the fundamental matrix of $x^{\prime }\left(t\right)=Q\left(t\right)x\left(t\right)$, and then let $r\left(\Phi_{Q}\left(t\right)\right)$ be the spectral radius of $\Phi_{Q}\left(t\right)$ [23]. The stability of disease-free periodic solution in the SILS model is proved by Floquet theorem and we assume that $\omega_{i}\;\left(i=1,\;2,\;\ldots \right)$ are the Floquet multipliers of Equation (18) [14]. Let the small perturbation of the disease-free periodic solution of the system be x(t)=(s(t),i(t),a(t),li(t),ls(t)), and linearize the approximation of system (2) and (3) to obtain the equations as follows

(15) $$x^{\prime }\left(t\right)=Q\left(t\right)x\left(t\right),\;t\neq nT,n\in N\;x\left(t^{+}\right)=Px\left(t\right),\;t=nT,n\in N$$

□

Hence, we can derive some matrices

(16) $$Q=\left[\begin{array}{cc} U & B\\ 0 & F-V \end{array}\right],\;P=\left[\begin{array}{cc} P1 & 0\\ 0 & P2 \end{array}\right]\;U=\left[\begin{array}{cc} -\left(\mu +\gamma \right) & 0\\ \mu & -\gamma \end{array}\right],\;B=\left[\begin{array}{cc} \alpha_{1}-\alpha_{2}\tilde{S}\left(t\right) & 0\\ 0 & 0 \end{array}\right]\;F=\left[\begin{array}{cc} \alpha_{2}\tilde{S}\left(t\right) & 0\\ 0 & 0 \end{array}\right],\;V=\left[\begin{array}{cc} \gamma +\mu +\alpha_{1} & 0\\ -\mu & \gamma \end{array}\right]\;P_{1}=\left[\begin{array}{cc} 1-C & 0\\ 0 & 1-C \end{array}\right],\;P_{2}=\left[\begin{array}{cc} 1+\frac{C\mu }{\gamma } & 0\\ 0 & 1-C \end{array}\right]$$

Since $\Phi_{Q}\left(t\right)$ is the fundamental matrix of $x^{\prime }\left(t\right)=Q\left(t\right)x\left(t\right)$, there exists $\dot{\Phi \left(t\right)}=\Phi_{\;}\left(t\right)Q\left(t\right)$, where $\Phi_{\;}\left(0\right)=E_{0}$ ($E_{0}$ is the identity matrix), obtained by Equation (12), Equation (14) and system (15), we can obtain the following matrix

(17) $$\Phi \left(t\right)=\left(\begin{array}{cc} e^{UT} & \Phi_{B}\left(t\right)\\ 0 & \Phi_{F-V}\left(t\right) \end{array}\right)$$

When t=nT, from Equation (17), we have

(18) $$P\Phi \left(t\right)=\left(\begin{array}{cc} P_{1}e^{UT} & P_{1}\Phi_{B}\left(t\right)\\ 0 & P_{2}\Phi_{F-V}\left(t\right) \end{array}\right)\;P_{1}e^{UT}=\left[\begin{array}{cc} \left(1-C\right)e^{-\left(\gamma +\mu \right)T} & 0\\ \left(1-C\right)e^{-\mu T} & \left(1-C\right)e^{-\gamma T} \end{array}\right]$$

We can infer the Floquet multipliers of Equation (18) as follows

(19) $$\omega_{1}=P_{1}e^{UT}\;\omega_{2}=P_{2}\Phi_{F-V}\left(t\right)$$

According to Floquet theorem, the disease-free periodic solution is locally asymptotically stable if |*ω_i_*| < 1, where *i* = 1, 2. Therefore, we define thresholds

(20) $$R_{0}≜\left(P_{2}\Phi_{F-V}\left(t\right)\right)$$

According to Floquet theorem, when $R_{0}<1$, the disease-free periodic solution ($\tilde{S}$,0,0,0,$\tilde{LS}$) of Equation (4) is locally asymptotically stable.

**Theorem** **2.** *When* $\alpha_{1}-\alpha_{2}S\left(t\right)<0$ *and* $R_{0}<1$ *, the disease-free periodic solution of the system is global asymptotic stability.*

**Proof.**  For WRSNs, we expect the number of nodes to be greater than or equal to the initial number of nodes in the stable state. Based on Equations (1a)–(1f), we define the condition as follows

(21) Λ−γN(t)≥0

□

The following system (22) can be obtained from Equations (2) and (3)

(22) $$\{\begin{array}{c} \begin{array}{c} S\left(t^{+}\right)=S\left(t\right)+CLS\left(t\right)\\ LS\left(t^{+}\right)=\left(1-C\right)LS\left(t\right) \end{array}\}\;\;\;t=nT\left(n=1,\;2,\;3\ldots \right)\\ \begin{array}{c} \frac{dS\left(t\right)}{dt}\leq \Lambda -\left(\mu +\gamma \right)S\left(t\right)\\ \frac{dLS\left(t\right)}{dt}\leq -\left(\mu +\gamma \right)LS\left(t\right)+\frac{\Lambda \mu }{\gamma } \end{array}\}\;t\neq nT\left(n=1,\;2,\;3\ldots \right) \end{array}$$

Based on system (22), we consider the following comparison system as follows

(23) $$\{\begin{array}{c} \begin{array}{c} x_{1}\left(t^{+}\right)=x_{1}\left(t\right)+Cx_{2}\left(t\right)\\ x_{2}\left(t^{+}\right)=\left(1-C\right)x_{2}t \end{array}\}\;\;t=nT\left(n=1,\;2,\;3\ldots \;\right)\\ \begin{array}{c} x_{1}^{\prime }t=\Lambda -\left(\mu +\gamma \right)x_{1}\left(t\right)\\ x_{2}^{\prime }t=-\left(\mu +\gamma \right)x_{2}\left(t\right)+\frac{\Lambda \mu }{\gamma } \end{array}\}\;t\neq nT\left(n=1,\;2,\;3\ldots \right) \end{array}$$

According to the impulse differential equation comparison theorem [24], we can obtain some inequalities as follows

(24) $$St\leq x_{1}\left(t\right)\;LS\left(t\right)\leq x_{2}\left(t\right)$$

When t→∞, $x_{1}\left(t\right)\to S\left(t\right)\;,x_{2}\left(t\right)\to LS\left(t\right)$. Moreover, there exists a positive ε, for any $t>t_{1}>0$, there exists some inequalities as follows

(25) $$S\left(t\right)\leq x_{1}\left(t\right)<\tilde{S}+\varepsilon \;LS\left(t\right)\leq x_{2}\left(t\right)<\tilde{LS}+\varepsilon$$

Equation (26) can be obtained from the second and third equations of Equations (2) and (3)

(26) $$\{\begin{array}{c} \begin{array}{c} \frac{dI\left(t\right)}{dt}\leq \left[-\alpha_{1}-\mu -\gamma +\alpha_{2}\left(\tilde{S}+\;\varepsilon \right)\right]I\left(t\right)\\ \frac{dLI\left(t\right)}{dt}\leq -\gamma LI\left(t\right)+\mu I\left(t\right) \end{array}\}\;t\neq nT\left(n=1,\;2,\;3\ldots \;\right)\\ \begin{array}{c} I\left(t^{+}\right)=\left(1+\frac{C\mu }{\gamma }\right)I\left(t\right)\\ LI\left(t^{+}\right)=\left(1-C\right)LI\left(t\right) \end{array}\}\;\;\;\;\;\;\;t=nT\left(n=1,\;2,\;3\ldots \right) \end{array}$$

According to the comparison theorem, we have $u_{1}\left(t\right)\leq I\left(t\right)$, $u_{2}\left(t\right)\leq LI\left(t\right)$, and construct the following system, where $\left(u_{1}\left(t\right),u_{2}\left(t\right)\right)$ is the solution to Equation (26).

(27) $$\{\begin{array}{c} u^{\prime }\left(t\right)=\left(F-V\right)u\left(t\right)\;\;\;\;t\neq nT\left(n=1,\;2,\;3\ldots \right)\\ \begin{array}{c} u_{1}\left(t^{+}\right)=\left(1+\frac{C\mu }{\gamma }\right)u_{1}\left(t\right)\\ u_{2}\left(t^{+}\right)=\left(1-C\right)u_{2}\left(t\right)\; \end{array}\}\;t=nT\left(n=1,\;2,\;3\ldots \right) \end{array}$$

The solution to Equation (27) can be expressed as follows

(28) $$u\left(u_{1},u_{1}\right)=\Phi_{F-V}\left(t-nT\right)u\left(nT^{+}\right)\;$$

When t=nT, $u\left(\left(n+1\right)T^{+}\right)=P_{2}\Phi_{F-V}\left(t-nT\right)u\left(nT^{+}\right)$. When $R_{0}<1$ with t goes to infinity, it exists $u_{1}\to 0$ and $u_{2}\to 0$ so we have

(29) $$\underset{t\to \infty }{\lim}\;I\left(t\right)=0\;\underset{t\to \infty }{\lim}LI\left(t\right)=0$$

Hence, at any time $t>t_{2}>t_{1}$, existing

(30) $$0<I\left(t\right)<\varepsilon_{2}\;0<LI\left(t\right)<\varepsilon_{2}$$

Equation (31) can be obtained from the first and fourth equations of Equations (2) and (3)

(31) $$\{\begin{array}{c} \begin{array}{c} \Lambda -\left(\alpha_{2}\varepsilon_{2}+\mu +\gamma \right)S\left(t\right)\leq \frac{dS\left(t\right)}{dt}\leq \Lambda -\left(\mu +\gamma \right)S\left(t\right)\\ \left(\gamma +\mu \right)S\left(t\right)-\Lambda \leq \frac{dLS\left(t\right)}{dt}\leq -\left(\mu +\gamma \right)LS\left(t\right)+\frac{\Lambda \mu }{\gamma } \end{array}\}\;t\neq nT\left(n=1,\;2,\;3\ldots \right)\\ \begin{array}{c} \;S\left(t^{+}\right)=S\left(t\right)+CLS\left(t\right)\\ LS\left(t^{+}\right)=\left(1-C\right)LS\left(t\right) \end{array}\}\;\;\;\;\;\;\;\;\;\;\;t\neq nT\left(n=1,\;2,\;3\ldots \right)\; \end{array}$$

In order to use the comparison theorem, transformation to Equation (31) is as follows

(32) $$\{\begin{array}{c} \begin{array}{c} y_{1}^{\prime }t=\Lambda -\left(\alpha_{2}\varepsilon_{2}+\mu +\gamma \right)y_{1}\left(t\right)\\ y_{2}^{\prime }t=\left(\gamma +\mu \right)y_{1}\left(t\right)-\Lambda \end{array}\}\;t\neq nT\left(n=1,\;2,\;3\ldots \right)\\ \begin{array}{c} y_{1}\left(t^{+}\right)=y_{1}\left(t\right)+Cy_{2}\left(t\right)\;\\ y_{2}\left(t^{+}\right)=\left(1-C\right)y_{2}t \end{array}\}\;\;\;t=nT\left(n=1,\;2,\;3\ldots \right) \end{array}$$

Equation (32) has a set of positive solutions $\tilde{y}=\left({\tilde{y}}_{1},{\tilde{y}}_{2}\right),$ and $\underset{\varepsilon_{2}\to 0}{\lim}\tilde{y}=\left(\tilde{S},\tilde{LS}\right)$. By comparing the theorem of differential equations of impulses, we can obtain the inequality group of Equation (33) as follows

(33) $$y_{1}\left(t\right)<S\left(t\right)<x_{1}\left(t\right)\;y_{2}\left(t\right)<LS\left(t\right)<x_{2}\left(t\right)$$

As t tends to infinity, we have

(34) $$y_{1}\to {\tilde{y}}_{1}\;x_{1}\to \tilde{S}\;y_{2}\to {\tilde{y}}_{2}\;x_{2}\to \tilde{LS}$$

At any $t>t_{3}>t_{2}$, when $\varepsilon_{3}>0$, we have

(35) $${\tilde{y}}_{1}-\varepsilon_{3}<S\left(t\right)<\tilde{S}+\varepsilon_{3}\;{\tilde{y}}_{2}-\varepsilon_{3}<LS\left(t\right)<\tilde{LS}+\varepsilon_{3}$$

When t tends to infinity, $S\left(t\right)\to \tilde{S},LS\left(t\right)\to \tilde{LS}$. Thus, Theorem 2 is proved.

## 4. Persistence of Malware Transmission

In this section, the persistence of malware transmission is the focus of our discussion. If the system meets certain conditions, the spread of malware in the WRSNs will continue, which is known as the persistence of spreading malware.

**Lemma** **1.** *There exists* δ>0 *such that the solution verifies the system of inequalities as follows, when* $\left(\alpha_{1}-\alpha_{2}S\right)<0$ *and* $R_{0}>1$

(36) $$\{\begin{array}{c} \underset{t\to \infty }{lim}sup\;I\left(t\right)>\delta \;\\ \underset{t\to \infty }{lim}\sup\;LI\left(t\right)>\delta \; \end{array}\;$$

**Proof.**  In order to make WRSNs work normally, we want the number of nodes to be larger than the initial number when the system is stable. Therefore, according to Equation (1f), we can obtain the following relation.

(37) Λ−γN(t) ≥0 

□

Using proof by contradiction, if the above conclusion is not valid, there is a time variable $t_{1}>0$. For any time $t>t_{1}$, we have I(t)<δ and LI(t)<δ. Based on Equations (2) and (3), the following system can be written as follows

(38) $$\{\begin{array}{c} \begin{array}{c} S^{\prime }\left(t\right)\geq \Lambda +\left[\alpha_{1}-\alpha_{2}{}_{\;}S\left(t\right)\right]\delta -\left(\mu +\gamma \right)S\left(t\right)\\ LS^{\prime }\left(t\right)\geq \left(\mu +\gamma \right)S\left(t\right)-\Lambda \end{array}\}\;\;\;\;\;\;\;\;t\neq nT\left(n=1,\;2,\;3\ldots \right)\\ \begin{array}{c} S\left(t^{+}\right)=S\left(t\right)+CLS\left(t\right)\\ LS\left(t^{+}\right)=\left(1-C\right)LS\left(t\right) \end{array}\}\;\;\;\;\;\;\;\;\;\;\;\;\;\;\;\;\;\;\;\;\;\;\;\;\;\;\;t=nT\left(n=1,\;2,\;3\ldots \right) \end{array}$$

Based on the above system, the following system will be obtained

(39) $$\{\begin{array}{c} \begin{array}{c} Z_{1}^{'}\left(t\right)=\Lambda +\left[\alpha_{1}-\alpha_{2}{}_{\;}S\left(t\right)\right]\delta -\left(\mu +\gamma \right)Z_{1}\left(t\right)\\ Z_{2}^{'}\left(t\right)=\left(\mu +\gamma \right)Z_{1}\left(t\right)-\Lambda \end{array}\}\;t\neq nT\left(n=1,\;2,\;3\ldots \right)\\ \begin{array}{c} Z_{1}\left(t^{+}\right)=Z_{1}\left(t\right)+CZ_{2}\left(t\right)\\ Z_{2}\left(t^{+}\right)=\left(1-C\right)Z_{2}\left(t\right) \end{array}\}\;\;\;\;\;\;\;\;t=nT\left(n=1,\;2,\;3\ldots \right) \end{array}$$

The following conclusions can be inferred from the comparison theorem

(40) $$\{\begin{array}{c} S\left(t\right)\geq Z_{1}\left(t\right)\\ LS\left(t\right)\geq Z_{2}\left(t\right) \end{array}\;$$

Equation (39) has a positive periodic solution $\tilde{Z}=({\tilde{Z}}_{1},{\tilde{Z}}_{2})$, which is globally asymptotically stable and $\underset{\delta \to 0}{lim}\tilde{Z}=\left(\tilde{S},\tilde{LS}\right)$. There’s a positive number $\delta_{1}$, and for any $\delta_{1}>\delta$, we have ${\tilde{Z}}_{1}\geq \tilde{S}-\varepsilon_{1}$ and ${\tilde{Z}}_{2}\geq \tilde{LS}-\varepsilon_{1}$. By the comparison theorem, there is a time variable $t_{2}>t_{1}$, and we set $\varepsilon_{2}$ to be positive. At any time $t>t_{2}$, there are inequalities as follows

(41) $$\{\begin{array}{c} S\geq Z_{1}\geq {\tilde{Z}}_{1}-\varepsilon_{2}\geq S-\varepsilon_{1}-\varepsilon_{2}\\ LS\geq Z_{2}\geq {\tilde{Z}}_{2}-\varepsilon_{2}\geq LS-\varepsilon_{1}-\varepsilon_{2} \end{array}$$

By combining Equations (2) and (3) with the relationship mentioned above we can obtain the following system

(42) $$\{\begin{array}{c} \begin{array}{c} I^{\prime }\left(t\right)\geq \left(-\alpha_{1}-\mu -\gamma +\alpha_{2}\left(\tilde{S}-\varepsilon_{1}-\varepsilon_{2}\right)\right)I\left(t\right)\\ LI^{\prime }\left(t\right)\geq \left(\mu +\gamma \right)I+\gamma \left(\tilde{LS}-\varepsilon_{1}-\varepsilon_{2}\right)-\Lambda \end{array}\}\;t\neq nT\left(n=1,\;2,\;3\ldots \right)\\ \begin{array}{c} I\left(t^{+}\right)=I\left(t\right)+CLI\left(t\right)\\ LI\left(t^{+}\right)=\left(1-C\right)LI\left(t\right) \end{array}\}\;\;\;\;\;\;\;\;\;\;t=nT\left(n=1,\;2,\;3\ldots \right) \end{array}$$

As $\varepsilon_{1}$ and $\varepsilon_{2}$ approach 0, the above inequality can be reduced to the following expression

(43) $$\{\begin{array}{c} \begin{array}{c} I^{\prime }\left(t\right)\geq \left(-\alpha_{1}-\mu -\gamma +\alpha_{2}\tilde{S}\right)I\left(t\right)\\ LI^{\prime }\left(t\right)\geq \left(\mu +\gamma \right)I+\gamma \tilde{LS}-\Lambda \end{array}\}\;\;\;\;\;\;\;\;t\neq nT\left(n=1,\;2,\;3\ldots \right)\\ \begin{array}{c} I\left(t^{+}\right)=I\left(t\right)+CLI\left(t\right)\\ LI\left(t^{+}\right)=\left(1-C\right)LI\left(t\right) \end{array}\}\;\;\;\;\;\;\;\;\;\;\;\;\;\;\;\;\;\;\;\;\;\;\;\;\;\;\;\;\;\;t=nT\left(n=1,\;2,\;3\ldots \right) \end{array}$$

Let $u\left(t\right)=\left(\begin{array}{c} u_{1}\left(t\right)\\ u_{2}\left(t\right) \end{array}\right)=\left(\begin{array}{c} I\left(t\right)\\ LI\left(t\right) \end{array}\right)$, we set up the system as follows

(44) $$\{\begin{array}{c} u^{\prime }\left(t\right)=\left(F-V\right)u\left(t\right)\;\;\;\;\;\;\;\;t\neq nT\left(n=1,\;2,\;3\ldots \right)\\ \begin{array}{c} u_{1}\left(t^{+}\right)=u_{1}\left(t\right)+Cu_{2}\left(t\right)\\ u_{2}\left(t^{+}\right)=\left(1-C\right)u_{2}\left(t\right) \end{array}\}\;\;\;\;t=nT\left(n=1,\;2,\;3\ldots \right) \end{array}$$

The above system satisfies $u\left(t,nT,u\left(nT^{+}\right)\right)=\phi_{F-v}\left(t-nT\right)u\left(nT^{+}\right)$, $U\left(\left(n+1\right)T^{+}\right)=P_{2}\phi_{F-v}\left(t-nT\right)u\left(nT^{+}\right)$. When $\left(\alpha_{1}-\alpha_{2}S\right)<0$ and *R*_0_ > 1, there is a time variable $t_{\;}>0.$ As t→∞, $u_{1}\to \infty$ and $u_{2}\to \infty$, we can draw the conclusions as follows

(45) $$\{\begin{array}{c} \underset{t\to \infty }{\lim}I=\infty \\ \underset{t\to \infty }{\lim L}I=\infty \end{array}\;$$

The above conclusion is in contradiction with the condition established previously. Therefore, Lemma 1 is proved.

**Theorem** **3.** *There exists positive integer η such that the solution verifies the system of inequalities as follows, when $\left(\alpha_{1}-\alpha_{2}S\right)<0$ and $R_{0}>1$*

(46) $$\{\begin{array}{c} \underset{t\to \infty }{lim}inf\;I\left(t\right)>\eta \\ \underset{t\to \infty }{lim}inf\;LI\left(t\right)>\eta \end{array}\;$$

From Lemma 1, there are two possible situations when the malware continues to spread as follows:

(a) When the time variable T is large enough, I(t)>η, LI(t)>η;
(b) When the time variable T is large enough, I(t) and LI(t) oscillate around η.

If Scenario (a) is true, the persistence of malware transmission is obvious, and we will focus our discussion on Scenario (b). Inequalities (47) can be obtained from Lemma 1, and in the case of oscillation, the relationship exists as follows

(47) $$\{\begin{array}{c} \begin{array}{c} I\left(t_{1}\right)\geq \delta \\ LI\left(t_{1}\right)\geq \delta \end{array}\}\;\;\;\;\;\;\;\;\;\;\;t_{1}\in \left(n_{1}T,\left(n+1\right)T\right]\\ \begin{array}{c} I\left(t_{2}\right)\geq \delta \\ LI\left(t_{2}\right)\geq \delta \end{array}\}\;\;\;\;\;\;\;\;\;\;\;\;t_{2}\in \left(n_{2}T,\left(n+1\right)T\right] \end{array}$$

Where $n_{2}>n_{1}$, when $t\in \left[t_{1},t_{2}\right]$, the relationship exists as follows

(48) $$\{\begin{array}{r} LI^{\prime }\left(t\right)=-\gamma LI\left(t\right)+\mu I\left(t\right)\geq -\gamma LI\left(t\right),\;t\neq nT,n\in N\\ LI\left(t^{+}\right)=\left(1-C\right)LI\left(t\right),\;t=nT,n\in N \end{array}$$

Thus available

(49) $$LI\left(t\right)\geq {\left(1-C\right)}^{n_{2}-n_{1}}LI\left(t_{1}\right)e^{-\gamma \left(t-t_{1}\right)}\geq {\left(1-C\right)}^{n_{2}-n_{1}}\delta e^{-\gamma \left(t-t_{1}\right)}\geq {\left(1-C\right)}^{n_{2}-n_{1}}\delta e^{-\gamma \left(n_{2}+1-n_{1}\right)}\;$$

Similarly, for I, the relationship exists as follows

(50) $$\{\begin{array}{r} I^{\prime }\left(t\right)=\left[-\alpha_{1}-\mu -\gamma +\alpha_{2}S\left(t\right)\right]I\left(t\right)\geq \left(-\alpha_{1}-\mu -\gamma \right)I\left(t\right),\;t\neq nT,n\in N\\ I\left(t^{+}\right)=I\left(t\right)+CLI\left(t\right),\;t=nT,n\in N \end{array}\;$$

By combining Equations (48) and (50), we obtain

(51) $$I\left(t\right)\geq \delta e^{-\left(\alpha_{1}-\mu -\gamma \right)\left(t-t_{1}\right)}+C^{n_{2}-n_{1}}{\left(1-C\right)}^{n_{2}-n_{1}}\delta e^{-\gamma \left(n_{2}+1-n_{1}\right)T}\;$$

Let $\eta =\min\{\delta e^{-\left(\alpha_{1}-\mu -\gamma \right)\left(t-t_{1}\right)}+C^{n_{2}-n_{1}}{\left(1-C\right)}^{n_{2}-n_{1}}\delta e^{-\gamma \left(n_{2}+1-n_{1}\right)T}$, ${\left(1-C\right)}^{n_{2}-n_{1}}\delta e^{-\gamma \left(n_{2}+1-n_{1}\right)}\}$ and $n_{2}-n_{1}\geq 0$. Just because $n_{2}-n_{1}$ is bounded, η cannot be infinitesimal. Thus, we can derive $I\left(t\right)\geq \eta_{1}$ and $LI\left(t\right)\geq \eta_{1}$. Similarly, for $t>t_{2}$, there is also a positive number $\eta_{2}$. 

In this way, we can obtain the sequence $\left\{\eta_{j}\right\},\;j=1,\;2,\cdots k\cdots$, this sequence can be represented as follows

(52) $$\{\begin{array}{c} I\left(t\right)\geq \eta_{k}>0\\ LI\left(t\right)\geq \eta_{k}>0 \end{array}$$

Let $\eta^{*}=\min\eta_{j}$, for any $t>t_{1}$, we have $I\left(t\right)\geq \eta^{*}>0$ and $LI\left(t\right)\geq \eta^{*}>0$. Therefore, Theorem 3 is proved.

## 5. Numerical Simulation

In this section, several numerical simulations are given to illustrate the correctness of the above theory. In fact, in order to reflect the characteristics and advantages of the pulse charging model, we make comparisons among the pulse charging model, the continuous charging model and the non-charging model. Among them, the model of continuous charging is derived from paper [21]. In Section 5.1, the malware-free T-period solution of SILS-P is obtained and verified, while the global stability of the other two charging models are presented. In Section 5.2, we analyze the impact of different variables on the threshold in different models. All of the simulations are based on DESKTOP-VEF0OI5 (Intel Core i5, 2.30 GHz) and MATLAB 2016a.

### 5.1. The Global Stability of the Disease-Free Equilibrium Solution

This subsection aims to verify Theorem 1 and Theorem 2 and compares three charging models when $R_{0}<1$. 

In reality, different distribution modes of nodes will affect the communication coverage area of nodes. The larger the communication coverage area is, the corresponding energy consumption will also increase [25]. However, the simulation in this section ignores the differences of communication mechanism and node distribution, and focuses on the change in the number of nodes. The number curve of nodes under the disease-free periodic solution can be realized by setting the relevant parameters in Table 2.

In Section 2, we put forward the pulse charging strategy, which can keep WSNs running permanently under certain circumstances. Through numerical simulation, the change of the number of nodes can be intuitively reflected, and the number of high-energy nodes can reflect the operating state to some extent.

In Section 3, the stability of the periodic solution was proved. As *t* goes to infinity, there will be no spread of malware in WSNs, and the function of WSNs will depend on the number of remaining high-energy nodes. When *t* tends to infinity, the number of high-energy nodes can reflect the effect of charging strategy to some extent.

Here, we assume that some parts of the low-energy nodes are charged in a cycle and some nodes will be charged in the next cycle. In order to reflect the pulse charging strategy and obtain a sufficiently small duty cycle, we consider charging as an instantaneous behavior. In addition, the far-field charging efficiency is low [22], and for the sake of being closer to the real physical environment, the charging rate of nodes C is set as a small constant.

The WSNs is assumed to have N=20 sensor nodes [26] and we suppose the following parameter $\Lambda \;=0.1,\;\gamma \;=0.005,\;\mu \;=0.05,\;\alpha_{2}=0.001,\;\alpha_{1}=0.01,\;C\;=0.05,\;T\;=10$ and the initial condition S(0)=18, I(t)=2, LI(0)=0, LS(0)=0. Thus, when t→∞, whether *R*_0_ < 1 or $R_{0}>1$, the whole number of sensor nodes is constant at 20 (i.e., S(t)+I(t)+LS(t)+LI(t)≤20), comprising the initial value (i.e., S(t)+I(t)+LS(t)+LI(t)=20). It is assumed that $R_{00}$ is the threshold in the continuous charging model and $R_{000}$ is the threshold of the non-charging model. 

**Figure 1:**
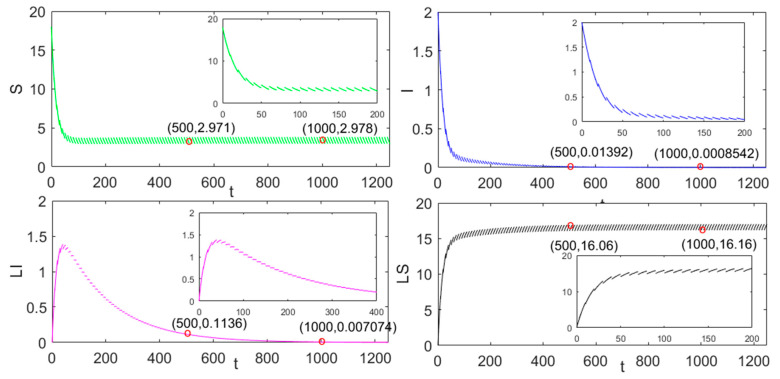
The global stability of the disease-free periodic solution $\left(\tilde{S},0,0,0,\tilde{LS}\right)$ in the pulse charging model.

From Equation (20) and the value of the previous parameters of SILS-P, we can calculate $R_{0}=0.9037<1$. From Theorem 2, the disease-free periodic solution ($\tilde{S}$,0,0,0,$\tilde{LS}$) is globally stable, as presented in Figure 1. Moreover, it is noted that S(t=500)=2.971, S(t=1000)=2.978; I(t=500)=0.01392, I(t=1000)=0.0008542; LI(t=500)=0.1136, LI(t=1000)=0.007074; LS(t=500)=16.06, LS(t=1000)=16.16. 

It is obvious that the values of S and LS nodes tend to be stable, this characteristic conforms to Equation (35) in Theorem 2. In the same way, the values of I and LI nodes almost disappear with the increase of t which conforms to Equation (29) in Theorem 2. Then, with the same value of parameters, the other two charging models are compared. It is noted that if $R_{00}=\frac{\alpha_{2}\Lambda {\left(C+\gamma \right)}^{2}}{\left[\left(C+\gamma \right)\left(\mu +\gamma \right)-C\mu \right]\left[\left(\alpha_{1}+\mu +\gamma \right)\left(C+\gamma \right)-C\mu \right]}\;$ and $R_{000}=\frac{\alpha_{2}\Lambda \gamma^{2}}{\gamma^{2}\left(\mu +\gamma \right)\left(\alpha_{1}+\mu +\gamma \right)}$ [21], we can obtain $R_{00}=0.5360$ and $R_{000}=0.0280$. 

**Figure 2:**
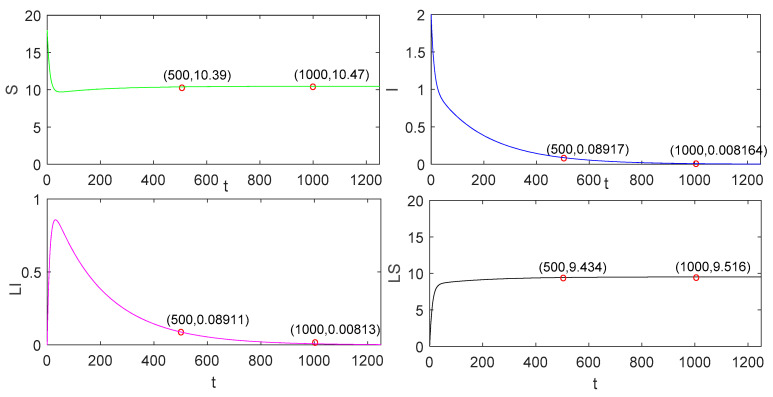
The global stability of the disease-free equilibrium solution in the continuous charging model.

**Figure 3:**
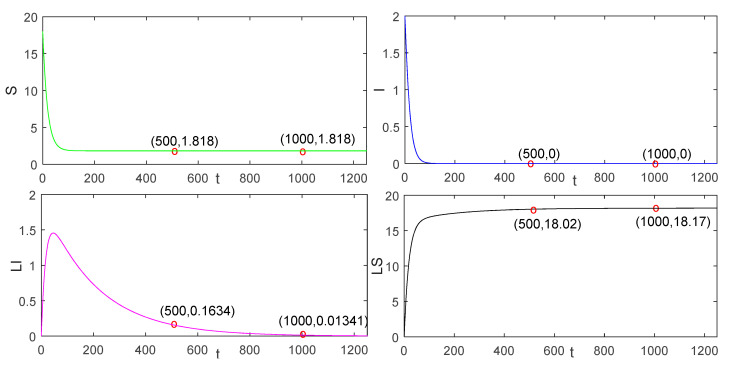
The global stability of the disease-free equilibrium solution in the non-charging model.

As shown in Figure 2 and Figure 3, we discover that the number of S nodes is 10.47 when S tends to be stable in the continuous charging model. In the pulse charging model, the number of S nodes is 2.978 when S tends to be stable. When the non-charge policy is implemented, the number of S nodes is 1.818. From Figure 2 and Figure 3, we can also find out the characteristics of the global stability in Theorem 3 in paper [21].

Apparently, it is easy to recognise that the number of S nodes is the largest in the continuous charging model, followed by the pulse charging model, and, finally, the non-charging model. Therefore, this phenomenon suggests that the continuous charging is the most efficient, followed by the pulse charging model, and, finally, the non-charging model. However, compared with the continuous charging model in [21], because of the short time slot of charging behavior, our model is more scientific and realistic. We observe that the time at which I converges to zero with the pulse charging model is shorter than that with the traditional continuous charging model. In this respect, the advantage of the pulse charging model can be reflected.

### 5.2. Relations between the Threshold and Parameters

Accordingly, the effects of the parameters on $R_{0}$, $R_{00}$ and $R_{000}$ will be discussed as follows.

**Figure 4:**
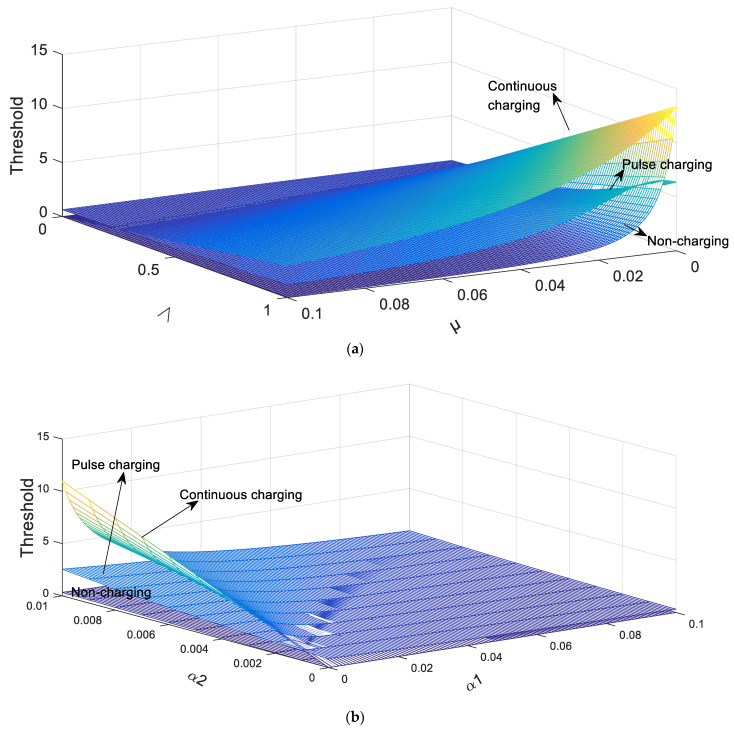
The relations between the threshold and parameters in three charging models. (**a**) The effect of Λ and μ on the threshold in three charging models. (**b**) The effect of $\alpha_{1}$ and $\alpha_{2}$ on the threshold in three charging models.

In Figure 4a, the parameters are set as $\gamma =0.005,\;\alpha_{2}=0.001,\;\alpha_{1}=0.01,\;C\;=0.05,\;T\;=10,\;\mu \in \left[0,0.1\right],\;\Lambda \in \left[0,1\right].$

As shown in Figure 4a, it is obvious that the larger Λ means the threshold larger and the larger μ means the threshold less. In addition, the value of the threshold in the pulse charging model is larger than that in the continuous charging model, but is smaller than that in the non-charging model in most cases. 

Besides, it is assumed that $\Lambda =0.1,\;\gamma =0.005,\;\mu =0.05,\;C=0.05,\;T=10,\;\alpha_{1}\in \left[0,0.1\right],\;\alpha_{2}\in \left[0,0.01\right]$ in Figure 4b. It is indisputable that the increase of $\alpha_{2}$ and the decrease of $\alpha_{1}$ contribute to the growth of the threshold. Furthermore, it is clear to see that the influence of $\alpha_{1}$ on the threshold is greater than that of $\alpha_{2}$. 

In addition, it can be seen from the Figure 4a,b that with the change of parameters, the rate of change of threshold of the pulse charging model is generally smaller than that of the continuous charging model.

## 6. Conclusions and Future Work

Based on the existing SILS model [21], this paper proposes a pulse charging method, which is more scientific and realistic. Then, the paper analyzes the WSNs under pulse charging, obtains the malware-free T-period solution and carries out the stability analysis. In addition, the persistent spread of malware is also discussed and the persistence of the disease is demonstrated. By comparing the cases $R_{0}<1$ and $R_{0}>1$, the dynamic of the malware spreading in WRSNs is revealed. The simulation results show that the malicious software will die out (malware-free T-period solution) or the malicious software will spread continuously (persistence of malware transmission). At the same time, the paper compares the relationship among the threshold and system parameters under three charging strategies: pulse charging, continuous charging and no charging.

In this paper, a pulse charging strategy is introduced in a homogeneous network. However, with the development of the Internet of Things (IOT) industry, heterogeneous network technology has become mainstream, and the pulse charging strategy for the heterogeneous network is one of our future research directions. Additonally, some impulsive models can be extended into depict the dynamics of infectious disease based on the security problem of WRSNs [15] in future.

## Figures and Tables

**Table 1:** Research on network security in WSNs.

Authors	Participants	Goal
Xiaotong Xu et al. [2]	Attack and defense based on evolutionary game theory	Obtain higher security benefits, more suitable for the actual situation of network attack and defense
Hongbin Wang et al. [3]	Sensor network node under the attack of Sybil	Accurate detection of Sybil attacks using RSSI
G. Shanmugavadivel et al. [4]	Data security in wireless body area networks (WBAN)	Based on AES and efficient task flow scheduling, an enhanced data security model using genetic GA is proposed
Liu Yang et al. [5]	Clustering security in industrial wireless sensor networks (IWSNS)	A cluster head selection method based on fuzzy theory is proposed to balance energy saving and safety
Monette H. Khadr et al. [6]	Data security in heterogeneous networks	A key selection algorithm for protecting data is proposed.
Abhilash Singh et al. [7]	Attack and defense in WSNs	An intrusion prevention method based on Gaussian Process Regression (GPR) model and machine learning is proposed
Deepti Singh et al. [8]	Attack and defense in wireless medical sensor networks (WMSNs)	This paper presents an elliptic curve cryptosystem (ECC) based on random prediction model
Ning Sun et al. [9]	Security of information transmission in WSNs	The key management and design technology of encryption technology are improved

**Table 2:** Description of the parameters.

Parameters	Interpretation	Units	Source
Λ	The birth rate of nodes	0.1	[21]
γ	The mortality rate of nodes	0.005	[21]
μ	The rate of transforming both the high-energy nodes I and S into the low-energy nodes LI and LS	0.05	[21]
$\alpha_{2}$	The data transfer coefficient	0.001	[21]
$\alpha_{1}$	The conversion rate of infected nodes become susceptible nodes	0.01	[21]
C	The charging rate of nodes	0.05	[21]
T	The period of pulse charging	10	Assumed
N	The whole number of sensor nodes	20	Assumed

## Data Availability

Not applicable.
